# Divergence in Eco-Physiological Responses to Drought Mirrors the Distinct Distribution of *Chamerion angustifolium* Cytotypes in the Himalaya–Hengduan Mountains Region

**DOI:** 10.3389/fpls.2016.01329

**Published:** 2016-08-31

**Authors:** Wen Guo, Jie Yang, Xu-Dong Sun, Guang-Jie Chen, Yong-Ping Yang, Yuan-Wen Duan

**Affiliations:** ^1^Key Laboratory for Plant Diversity and Biogeography of East Asia, Kunming Institute of Botany, Chinese Academy of SciencesKunming, China; ^2^Plant Germplasm and Genomics Center, Germplasm Bank of Wild Species, Kunming Institute of Botany, Chinese Academy of SciencesKunming, China; ^3^University of the Chinese Academy of SciencesBeijing, China; ^4^Institute of Tibetan Plateau Research at Kunming, Kunming Institute of Botany, Chinese Academy of SciencesKunming, China; ^5^School of Life Sciences, Yunnan Normal UniversityKunming, China; ^6^Key Laboratory of Plateau Lake Ecology and Global Change, School of Tourism and Geography, Yunnan Normal UniversityKunming, China

**Keywords:** *C. angustifolium*, cytotype distribution, common garden experiment, drought tolerance, physiological fitness

## Abstract

Polyploid species generally occupy harsher habitats (characterized by cold, drought and/or high altitude) than diploids, but the converse was observed for *Chamerion angustifolium*, in which diploid plants generally inhabit higher altitudes than their polyploid derivatives. Plants at high altitudes may experience cold-induced water stress, and we therefore examined the physiological responses of diploid and hexaploid *C. angustifolium* to water stress to better understand the ecological differentiation of plants with different ploidy levels. We conducted a common garden experiment by subjecting seedlings of different ploidy levels to low, moderate, and severe water stress. Fourteen indicators of physiological fitness were measured, and the anatomical characteristics of the leaves of each cytotype were determined. Both cytotypes were influenced by drought, and diploids exhibited higher fitness in terms of constant root:shoot ratio (R:S ratio) and maximum quantum yield of PS II (*F_v_/F_m_*), less reduced maximal photosynthetic rate (*A*_max_), transpiration rate (*E*), intercellular CO_2_ concentration (*C*_i_) and stomatal conductance (*g*_s_), and higher long-term water use efficiency (WUE_L_) under severe water stress than did hexaploids. Analysis of leaf anatomy revealed morphological adjustments for tolerating water deficiency in diploids, in the form of closely packed mesophyll cells and small conduits in the midvein. Our results indicate that diploid *C. angustifolium* is more tolerant of drought than hexaploid plants, ensuring the successful survival of the diploid at high altitudes. This eco-physiological divergence may facilitate the species with different cytotypes to colonize new and large geographic ranges with heterogeneous environmental conditions.

## Introduction

Polyploidy, the state of having more than two complete chromosome sets per nucleus, has played a key role in the evolution and diversification of the plant kingdom (Leitch and Bennett, 1997; Soltis et al., 2009). Polyploidization can be accompanied by considerable cytological, morphological, and physiological alterations, meaning that ecological requirements can differ significantly between diploids and their polyploid derivatives (Ramsey and Schemske, 2002; Soltis et al., 2014). This may result in different adaptations in the different cytotypes and consequently in habitat segregation (Levin, 2004; te Beest et al., 2011), and it has long been hypothesized that polyploids may be able to occupy harsher environments relative to diploids because of the advantages of polyploidy (Grant, 1981; Levin, 1983). Observations supporting this hypothesis suggest that polyploids are common in alpine regions, arctic areas (Brochmann et al., 2004) and other ecologically extreme environments (Ramsey, 2011; Manzaneda et al., 2012; Hao et al., 2013; McAllister et al., 2015). However, many examples suggest that the frequency of diploids also tends to increase with altitude (Hardy et al., 2000; Sonnleitner et al., 2010) or latitude (Ricca et al., 2008), indicating that diploids may be more tolerant of certain stressful conditions (Buggs and Pannell, 2007; Visser and Molofsky, 2015). These conflicting results suggest that polyploids may not necessarily occupy more extreme habitats than their diploid parents, but rather can be regarded as ‘fill-in’ taxa that occupy habitats which become available for them.

Knowledge about the geographical distributions of different cytotypes may offer insights into the mechanisms responsible for their spatial separation (Duchoslav et al., 2010). In high-altitude mountains, the growth and reproduction of plants may be strongly influenced by low temperatures (Angert, 2006) and cold-induced drought stress (physiological drought; Hammel, 1967; Pockman and Sperry, 1996; Zhu et al., 2000). As a consequence, plants growing in high-altitude regions have developed various mechanisms to enhance their drought tolerance (Ma et al., 2010, 2014; Yang and Miao, 2010). Polyploids usually have larger xylem conduits than diploids due to chromosome doubling effects (Graciano-Ribeiro and Nassar, 2012; Hao et al., 2013); these conduits confer high levels of water transport efficiency (Maherali et al., 2009) but may be vulnerable to cavitation under drought stress due to the inverse relationship between hydraulic conductance and protection against embolism (Piñol and Sala, 2000; Martínez et al., 2002). On the contrary, narrow xylem conduits tend to have fewer and smaller pit membrane pores to reduce the occurrence of air seeding under high xylem tension (Wheeler et al., 2005), thus they might offer a selective advantage for diploids at high altitudes by minimizing the risk cost associated with xylem embolism.

As one of the most important biodiversity hotspots at similar latitudes in the Northern Hemisphere (Wu and Wu, 1998), the Himalaya-Hengduan Mountains region (HHMs) contains over 20,000 species of vascular plants, and harbors very rich alpine flora with a profusion of endemic species (Wu, 1988; Li and Li, 1993). Although it has been proposed that polyploidy has played only a minor role in plant diversification in this region (Liu, 2004; Nie et al., 2005; Yuan and Yang, 2008), the frequency of polyploidy is relatively high in some genera occurring there, for example, *Buddleja* (Chen G. et al., 2007), *Rheum* (Liu et al., 2010), *Anaphalis* (Meng et al., 2014), *Meconopsis* (Xie et al., 2014), and *Ephedra* (Wu et al., 2016). Recent cytogeographical investigations have indicated the importance of cytotype distribution patterns and have revealed altitudinal segregation between different ploidy levels in the HHMs region (Li et al., 2010; Yu et al., 2010; Zhai et al., 2011; Liang et al., 2015). However, the mechanisms underlying cytotype distribution still remain unclear, especially with respect to the roles played by physiological endurance in ecological differentiation among different cytotypes.

*Chamerion angustifolium* L. Holub (Onagraceae) is widespread throughout the northern hemisphere. This species is an autopolyploid perennial, with diploid (2*n* = 36), tetraploid (2*n* = 72), and hexaploid (2*n* = 108) cytotypes (Mosquin, 1967; Chen J.R. et al., 2007). In the HHMs, the species generally occupies open and disturbed habitats, and it has been observed that diploid plants inhabit higher altitudes than polyploid plants, which is consistent with the patterns of distribution in north America (Mosquin, 1967; Husband and Schemske, 1998; Chen J.R. et al., 2007). Recent studies have suggested that the current distribution of diploid and tetraploid *C. angustifolium* across elevations may be the result of differences in physiological tolerances to drought or cold (Thompson et al., 2014) and the adaptation to native elevation of each cytotype (Martin and Husband, 2013). In the present study, we therefore aimed to quantitatively examine the responses of different cytotypes of *C. angustifolium* to drought stress, with an emphasis on testing the hypothesis that conduits might be narrower in diploid than in polyploid plants, a factor which could explain to a certain extent the vicarious distribution of this plant species along altitudinal gradients.

## Materials and Methods

### Collections of Material

In 2013, we obtained all seeds used in the present study from two open-pollinated populations on Baima Snow Mountain, Yunnan province. The pure diploid population was located on a site at a higher altitude (28°23’38″ N, 98°59’32″ E, 4160 m) than the pure hexaploid population (28°25’38″ N, 98°58’15″ E, 3560 m). In each population, mature fruits and a small amount of leaf tissue were collected from multiple maternal plants. All seeds and leaf tissues were brought to the laboratory at Kunming Institute of Botany within 36 h and kept at 4°C.

### Ploidy Determination

We used flow cytometry and root-tip squashes to examine the ploidy of maternal plants. Approximately 1 cm^2^ fresh leaf tissue was chopped in 1.5 ml of pre-chilled WPB buffer (0.2 mol/L Tris⋅HCl, 4 mmol/L MgCl_2_⋅6H_2_O, 2 mmol/L EDTA Na_2_⋅2H_2_O, 86 mmol/L NaCl, 10 mmol/L sodium metabisulfite, 1% PVP-10, 1% (v/v) Triton X-100, pH 7.5). Fresh leaves from an *Oryza sativa* L. inbred line were chopped for use as an external standard (C-value = 0.86 pg/2C). After filtration, centrifugation, re-suspension and storage in the dark at 4°C for 10 min staining with 150 μl propidium iodide, the resulting cell suspensions were analyzed using a FACS-Vantage flow cytometer following the manufacturer’s recommendations (Partec, Germany; Tian et al., 2011). The histograms were analyzed with the FlowMax software package (Version 2.8.2, Partec GmbH, Germany). The FL2-area parameter (integrated fluorescence) was used to quantify DNA content. To examine the relationship between DNA content and ploidy, we carried out chromosome counts on root tips from seeds germinated in a Petri dish containing moist filter paper (Liu et al., 2006).

After identifying the ploidy of each maternal plant, seeds were germinated in plug trays filled with homogeneous humus soil, and placed in a canopied and naturally lit glasshouse at Yunnan Normal University. For each ploidy level, 180 robust seedlings of similar size were transplanted into pots (two seedlings per pot) 1 month later. All pots contained the same weight of a uniform mixture comprising equal volumes of peat and perlite. Soil surfaces were covered with a small quantity (c. 40 g) of perlite to minimize evaporation. Pots were randomly positioned on a single glasshouse bench and watered every other day to maintain saturation for 2 months. At the rosette stage, leaf tissue was sampled from each plant and re-screened using flow cytometry to exclude any seedlings of other ploidy levels.

### Glasshouse Drought Experiments

After 2 months of growth, we began drought stress treatments. Mortality following transplantation reduced the sample sizes for diploids and hexaploids to 78 and 80 pots, respectively. Fifteen ‘empty’ pots were filled with the same amount of the same soil mixture to measure soil evaporation rates. The remaining pots of seedlings and the ‘empty’ pots were divided into low, moderate, and severe water stress treatments. Water stress was applied by watering to 80, 50, and 20% of maximum field capacity (FC). Soil water content was maintained at these levels by weighing the pots every 2 days, recording the amount of water loss and rewatering to the required water levels immediately. The experimental period commenced on July 9, 2014 (day t_1_), and continued until August 10, 2014 (day t_2_). During this period, no fertilizer was added to any pot. The sides of the glasshouse were always open for aeration throughout the experiment, so that the temperature inside the glasshouse was closely linked to the ambient outside temperature (Yang et al., 2014).

### Growth and Water Use

For growth and water use measurements, each pot was treated as a replicate, with its two seedlings being measured together. To estimate biomass increment during the experiment, 5 pots of each cytotype at the beginning of the experiment (t_1_) and 15 pots (five pots per treatment) at the end of the experiment (t_2_) were harvested. From each pot, the two seedlings were bulked together, and then divided into two parts: roots, and all aboveground parts including stems, leaves, and flowers. The total weight of each part was then determined after drying in an oven at 80°C for 48 h. The dry mass (dm) accumulated during the experimental period in the root [root dry mass (RDM)] and the aboveground parts dry mass (ADM) was calculated by subtracting dm per pot at day t_1_ from that at day t_2_, for each cytotype and treatment type, and dividing by two to convert from per pot to per plant. Total dry mass (TDM) was the sum of RDM and ADM, and the ratio of root to shoot (R:S ratio) was calculated by dividing RDM by ADM.

From both empty pots and those containing seedlings, water loss was measured as the difference between the weight of each pot just after watering and that just before the next watering event, 48 h later. These measurements were taken throughout the experimental period. Within each watering treatment, the amount of water transpired per pot per day for each cytotype was determined by deducting mean daily water loss per empty pot (evaporation) from mean daily water loss per pot with plants (evaporation plus transpiration). From this, total transpired water use (TWU) per plant was calculated as the total water transpired per pot between day t_1_ and day t_2_ divided by two.

Long-term water use efficiency (WUE_L_) per plant, defined as the ratio between biomass production and water consumption for transpiration, was calculated, for each cytotype and treatment, as TDM/TWU (Ma et al., 2010).

### Gas Exchange, Transpiration, and Chlorophyll Fluorescence

Five to six pots for each treatment per ploidy level were randomly selected to measure gas exchange characteristics. For each pot, the fifth leaf down from the top, which was fully opened and matured, was selected on one plant, and the maximum photosynthetic rate (*A*_max_), stomatal conductance (*g_s_*), intercellular CO_2_ concentration (*C*_i_) and transpiration rate (*E*) were measured simultaneously using a LI-6400 XT infrared gas-analyser (LI-Cor Inc., Lincoln, NE, USA) for that plant. Measurements were taken between 9:30 and 12:00 h during sunny weather. Light levels were maintained at 1600 μmol m^-2^ s^-1^ (light-saturation points were derived from light response curves determined before the experiment) using artificial light provided by an LI-6400-02B LED light source (LI-COR Biosciences). The external CO_2_ concentration was maintained at 400 μmol mol^-1^ using portable CO_2_*/*air mixture tanks whose output was controlled by a LI-6400-01 CO_2_ injector (LI-COR Biosciences). Temperature and relative humidity were maintained at 24–26°C and 23–29%, respectively. Due to the lanceolate leaves of *C. angustifolium* usually couldn’t cover the leaf chamber (6 cm^2^), leaves were cut and scanned using a Canon Scan Lide 110 after the measurements had been taken. Then the leaf areas were analyzed by Scion Image (Version 4.0.3, National Institutes of Health, USA) so that leaf gas exchange parameters could be calculated on a per area basis. Instantaneous water use efficiency (WUE_i_) was defined and calculated as *A*_max_/*E* (Sapeta et al., 2013).

Chlorophyll fluorescence parameters were measured between 6:00 and 7:00 h on leaves that had been dark adapted for 10 h. These measurements were taken on the same day as the leaf gas exchange measurements. The maximum quantum yield of photosystem II [PSII; *F*_v_*/F*_m_ = (*F*_m_-*F*_o_)*/F*_m_] was measured using a LI-6400-40 leaf chamber fluorometer (LI-COR Biosciences). Five plants in each treatment per cytotype were analyzed (Mena-Petite et al., 2000).

### Leaf Water Status

To determine leaf water status, leaves were collected from other five different plants in one treatment. One fully mature leaf of each selected plant was stored in plastic bag on wet tissues until required for measurement of leaf area. The method used for analyzing leaf area was as described in the gas exchange section. The fresh mass (fm) of the measured leaves was then determined. Before measuring the saturated mass (sm) of the leaves, we allowed them to become turgid by resting them in water for 1 h. Leaves were oven-dried at 80°C for 48 hours before dm was determined. Relative water content (RWC) was expressed as 100% [(fm-dm)/(sm-dm)] (Yamasaki and Dillenburg, 1999). Leaf dry mass per unit area (LMA) was also calculated.

### Leaf Anatomy

Leaves harvested from each of five different plants in one treatment were preserved in malondialdehyde (MDA). The 5 mm × 5 mm fragments from the adaxial side of the leaf and the midrib were used for embedding. The sample was incubated successively in the following solutions: 0.1 M PBS for 10 min (three times), 30% ethanol for 1 h, 50% ethanol for 1 h, 70% ethanol for 1 h, 80% ethanol for 1 h, 90% ethanol for 1 h, 95% ethanol for 1 h, 100% ethanol for 1 h, 100% ethanol:histoclear (1:1) for 4 h, histoclear for 1 h (two times), histoclear:paraffin (1:1) for 12 h (40°C), histoclear:paraffin (1:1) for 4 h (60°C), paraffin for 4 h (60°C), and paraffin for 12 h (60°C), and leaf tissues were then embedded in paraffin. Transverse cross-sections of the embedded samples were obtained with a microtome (Leica RM 2015) equipped with a freshly produced glass knife (Leica 819). The sections were then placed on glass slides.

For histochemical analyses, 12-μm-thick sections were stained with 1% Safranin for 12 h and 1% Fast Green for 10 s. In order to examine the lignified cell walls in midribs, 50-μm-thick sections were stained for 5 min with 1% phloroglucinol in 6 N HCl (Zhou et al., 2009). Stained cross sections were scanned using a brightfield microscopy (Leica DM 1000; Sun et al., 2013).

Leaf blade thickness, palisade parenchyma thickness, lacunar parenchyma thickness, leaf central vein diameter, xylem conduit diameter, leaf central vein total area, and xylem cell area were then measured from digital photographs with the Image J software (Version 1.45, National Institutes of Health, USA).

### Statistical Analyses

Data for all measured variables were analyzed using the general linear model (PROC GLM) to test the effects of cytotype, treatments, and their interactions. Significant differences among treatments for each cytotype were compared using one-way analysis of variance (ANOVA), and an independent-samples t test was used to compare the differences among cytotypes for each treatment. The homogeneity of variances was tested before analysis. Separate one-way ANOVAs were performed which assume independence between dependent variables and multiple traits measured for individual plants, but due to insufficient statistical power, correction of family-wise error rates for trait functional groups or individuals could not be performed. All statistical analyses were carried out using the SPSS statistical software package (Version 19.0, IBM, USA).

## Results

### Cytotype Composition

Flow cytometry analyses revealed two DNA ploidy levels in our samples: DNA-diploid (1.081 ± 0.009 pg/2C) and DNA-hexaploid (3.008 ± 0.084 pg/2C). The hexaploids thus had triple the DNA content of the diploids, a finding which was confirmed by chromosome counts.

### Plant Growth and Water Use Traits

**FIGURE 1:**
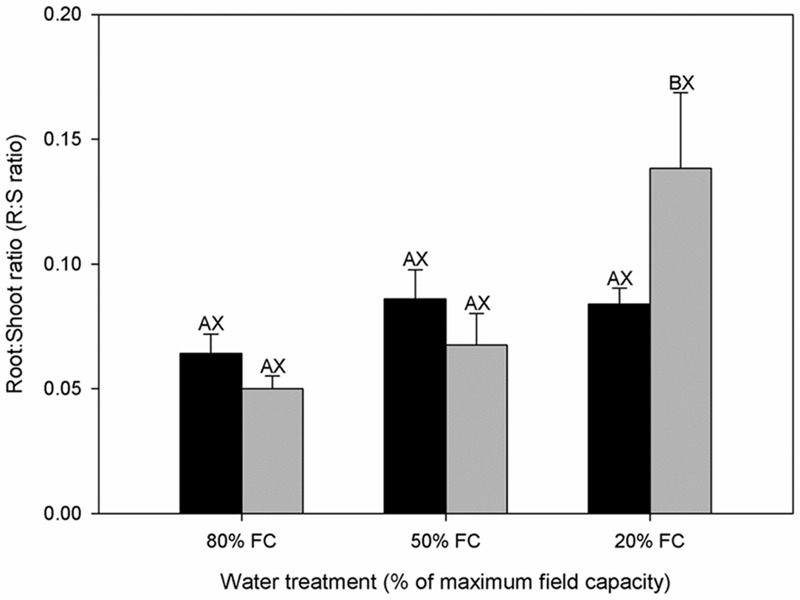
**Root:Shoot ratio (R:S ratio) of diploids (black bars) and hexaploids (gray bars) under different soil water conditions (80% of maximal field capacity (FC), 50% FC, 20% FC).** Each value is presented as mean ± SE. Letters distinguish between statistically separable (*p* < 0.05) values for different water treatments (A, B, C) and for different cytotypes (X, Y).

Diploids and hexaploids of *C. angustifolium* exhibited different biomass allocation strategies in response to drought stress, and diploids generally accumulated more biomass during the experiment across all water gradients than hexaploids. The differences between cytotypes were significant for TDM accumulation and ADM accumulation under high water stress, and for RDM accumulation under low water stress (**Table 1**). Although TDM apparently declined in both cytotypes as the available soil water decreased, both ADM and RDM decreased in diploids, whereas hexaploids only showed a reduction in ADM. Due to the difference in dry weight allocation, water stress did not result in a significant difference for the R:S ratio in diploids, but it caused a significant increase in hexaploids (**Figure 1**).

**Table 1:** Comparison of 11 measured indicators between *Chamerion angustifolium* diploids and hexaploids, across three different soil water treatments (80% of maximal field capacity (FC), 50% FC, and 20% FC).

	Water treatment (% of maximum FC)		
	Low stress	Medium stress	High stress
	
Variable and cytotype	80% FC	50% FC	20% FC
**Total dry mass (TDM) (g)**			
Diploid	2.016 ± 0.452 A,X	0.956 ± 0.082 B,X	0.558 ± 0.087 B,X
Hexaploid	1.086 ± 0.152 A,Y	0.784 ± 0.128 A,X	0.233 ± 0.061 B,Y
**Aboveground parts dry mass (ADM) (g)**			
Diploid	1.877 ± 0.428 A,X	0.866 ± 0.079 B,X	0.503 ± 0.076 B,X
Hexaploid	1.041 ± 0.150 A,Y	0.739 ± 0.118 A,X	0.170 ± 0.071 B,Y
**Root dry mass (RDM) (g)**			
Diploid	0.139 ± 0.025 A,X	0.091 ± 0.016 A,B,X	0.055 ± 0.012 B,X
Hexaploid	0.045 ± 0.006 A,Y	0.046 ± 0.016 A,Y	0.051 ± 0.013 A,X
**Maximal photosynthetic rate (*A*_max_) (μmol CO_2_ m^-2^ s^-1^)**			
Diploid	12.879 ± 0.528 A,X	10.407 ± 0.429 B,X	7.818 ± 0.752 C,X
Hexaploid	11.958 ± 0.495 A,X	9.828 ± 0.641 B,X	5.508 ± 0.318 C,Y
**Transpiration rate (*E*) (mmol H_2_O m^-2^ s^-1^)**			
Diploid	6.261 ± 0.314 A,X	7.043 ± 0.455 A,X	2.751 ± 0.192 B,X
Hexaploid	9.488 ± 0.189 A,Y	8.174 ± 0.460 B,X	2.385 ± 0.378 C,X
**Intercellular CO_2_ concentration (*C*_i_) (μmol CO_2_ mol^-1^)**			
Diploid	270.532 ± 7.727 A,X	294.814 ± 6.146 B,X	227.562 ± 6.860 C,X
Hexaploid	312.072 ± 2.703 A,Y	309.799 ± 4.578 A,X	233.299 ± 8.754 B,X
**Stomatal conductance (*g*_s_) (mmol H_2_O m^-2^ s^-1^)**			
Diploid	0.231 ± 0.016 A,X	0.223 ± 0.020 A,X	0.078 ± 0.005 B,X
Hexaploid	0.392 ± 0.010 A,Y	0.286 ± 0.026 B,X	0.058 ± 0.010 C,X
**Maximum quantum yield of PS II (*F*_v_*/F*_m_)**			
Diploid	0.816 ± 0.003 A,X	0.808 ± 0.005 A,X	0.808 ± 0.005 A,X
Hexaploid	0.812 ± 0.002 A,X	0.806 ± 0.004 A,X	0.770 ± 0.006 B,Y
**Total transpired water use (TWU) (kg)**			
Diploid	0.689 ± 0.169 A,X	0.358 ± 0.039 B,X	0.136 ± 0.021 B,X
Hexaploid	0.456 ± 0.049 A,X	0.309 ± 0.029 A,X	0.074 ± 0.007 B,Y
**Long-term water use efficiency (WUE_L_)**			
Diploid	2.887 ± 0.357 A,B,X	2.658 ± 0.090 A,X	4.193 ± 0.325 B,X
Hexaploid	2.316 ± 0.114 A,X	2.354 ± 0.229 A,X	3.149 ± 0.248 A,Y
**Leaf relative water content (RWC)**			
Diploid	0.898 ± 0.014 A,X	0.869 ± 0.005 A,X	0.790 ± 0.010 B,X
Hexaploid	0.873 ± 0.013 A,X	0.861 ± 0.009 A,X	0.843 ± 0.018 A,Y

*Each value represents mean ±*SE*. Letters after *SE* values distinguish between statistically separable (*P* < 0.05) values for different water treatments (A, B, C) and for different cytotypes (X, Y).*

**FIGURE 2:**
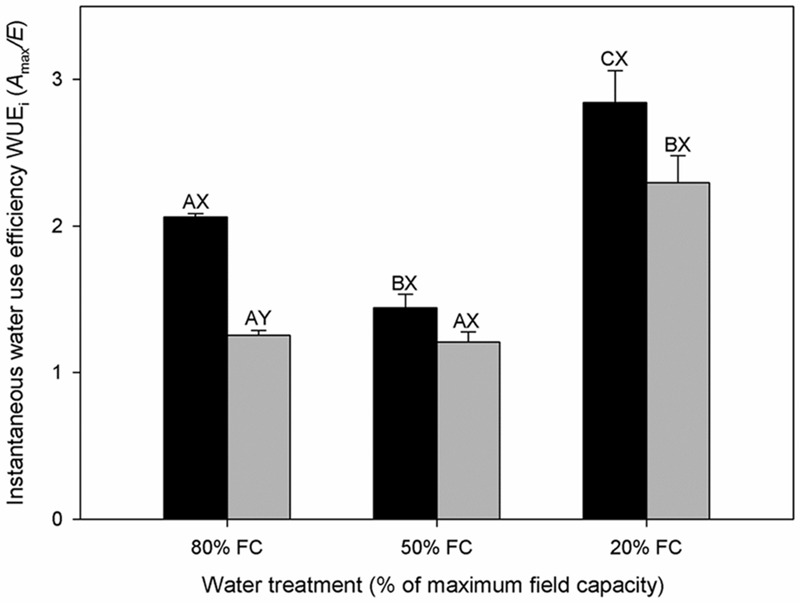
**Instantaneous water use efficiency (WUE_i_) of diploids (black bars) and hexaploids (gray bars) under different soil water conditions (80% of maximal FC, 50% FC, 20% FC).** Each value is presented as mean ± SE. Letters distinguish between statistically separable (*p* < 0.05) values for different water treatments (A, B, C) and for different cytotypes (X, Y).

Total TWU decreased significantly with decreasing soil water content in both cytotypes (**Table 1**). From low to severe water stress, diploids experienced a significant increase in WUE_L_ but hexaploids did not. Furthermore, diploids had higher WUE_L_ than hexaploids under severe water stress (**Table 1**). Transpiration rate (*E*) and WUE_i_ differed significantly among the three soil water contents (**Tables 1** and **2**). *E* decreased significantly with increasing water stress in both cytotypes, leading to a significant increase in WUE_i_ (**Figure 2**). Of the two ploidy levels, diploids had significantly lower *E* and higher WUE_i_ than hexaploids under low water stress.

**Table 2:** Comparisons of all variables measured in this experiment for diploid and hexaploid *C. angustifolium* seedlings.

Variable	Abb.	df	Watering treatment	df	Cytotypes	df	Treatment ^∗^ cytotypes interaction
Total dry mass	TDM	2	13.753^∗∗∗^	1	7.014^∗^	2	1.731
Aboveground parts dry mass	ADM	2	14.367^∗∗∗^	1	6.414^∗^	2	1.602
Root dry mass	RDM	2	3.143	1	13.702^∗∗^	2	4.143^∗^
Root:Shoot ratio	R:S ratio	2	6.713^∗^	1	0.358	2	3.752^∗^
Maximal photosynthetic rate	*A*_max_	2	56.42^∗∗∗^	1	8.134^∗∗^	2	1.413
Transpiration rate	*E*	2	146.417^∗∗∗^	1	21.749^∗∗∗^	2	13.339^∗∗∗^
Intercellular CO_2_ concentration	*C*_i_	2	72.102^∗∗∗^	1	15.541^∗∗^	2	4.154^∗^
Stomatal conductance	*g*_s_	2	124.880^∗∗∗^	1	26.736^∗∗∗^	2	15.760^∗∗∗^
Maximum quantum yield of PS II	*F*_v_*/F*_m_	2	20.554^∗∗∗^	1	20.126^∗∗∗^	2	12.763^∗∗∗^
Instantaneous water use efficiency	WUE_i_	2	51.247^∗∗∗^	1	25.894^∗∗∗^	2	2.535
Total transpired water use	TWU	2	4.064^∗^	1	2.762	2	0.344
Long-term water use efficiency	WUE_L_	2	16.818^∗∗∗^	1	5.877^∗^	2	1.538
Leaf relative water content	RWC	2	6.186^∗∗^	1	0.594	2	2.792
Leaf mass per unit area	LMA	2	6.690^∗∗^	1	12.862^∗∗^	2	0.269

*P-values are presented for watering treatment, cytotype, and their interaction.*

*^∗^*P* < 0.05; ^∗∗^*P* < 0.01; ^∗∗∗^*P* < 0.001.*

### Leaf Photosynthesis and Chlorophyll Fluorescence

Maximal photosynthetic rate (*A*_max_), intercellular CO_2_ concentration (*C*_i_) and stomatal conductance (*g*_s_) decreased significantly as water stress increased (**Table 1**), and there were significant differences between cytotypes in these three variables under specific drought stress treatments (**Tables 1** and **2**). Diploids had a higher value of *A*_max_ than hexaploids in all drought stress treatments and the difference was significant under 20% maximum FC (**Table 1**). The values of *C*_i_ and *g*_s_ were significantly higher in hexaploids than in diploids under low stress, whereas these differences were reduced and no significant difference could be detected under medium or high water stress (**Table 1**).

Maximum quantum yield of PS II (*F*_v_*/F*_m_) was similar between cytotypes for 80% FC and 50% FC, but it was significantly lower in hexaploids than in diploids under severe drought stress treatment (**Table 1**).

### Leaf Water Status and Histology

**FIGURE 3:**
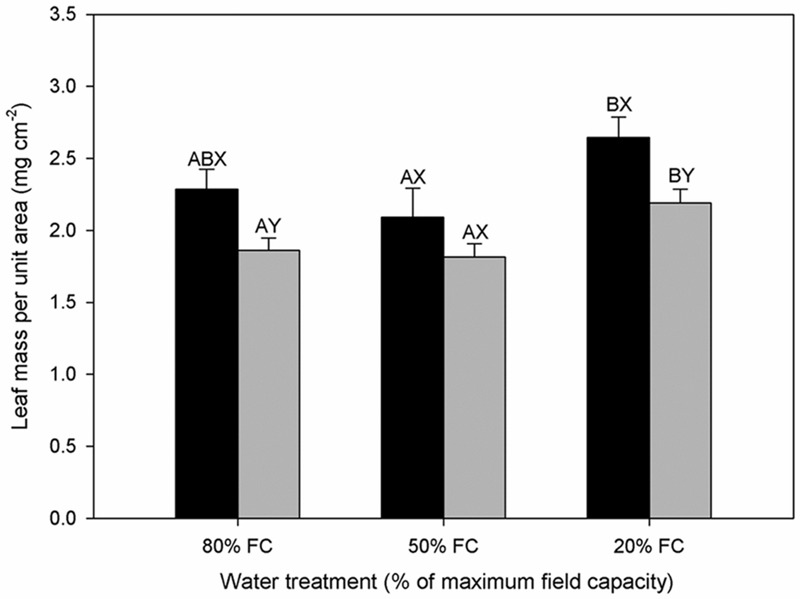
**Leaf mass per unit area (LMA) of diploids (black bars) and hexaploids (gray bars) under different soil water conditions (80% of maximal FC, 50% FC, 20% FC).** Each value is presented as mean ± SE. Letters distinguish between statistically separable (*p* < 0.05) values for different water treatments (A, B, C) and for different cytotypes (X, Y).

A significant decrease in leaf RWC was observed with increasing water stress for diploids, but there was no significant difference for hexaploids (**Table 1**). The value of RWC was significantly lower in diploids than in hexaploids under 20% FC. Leaf mass per unit area (LMA) varied significantly among water stress treatments and between ploidy levels. Drought stress gave rise to the increase in LMA from 50% FC to 20% FC for both cytotypes, but diploids showed significantly higher LMA than hexaploids under 80% FC and 20% FC (**Figure 3**).

**FIGURE 4:**
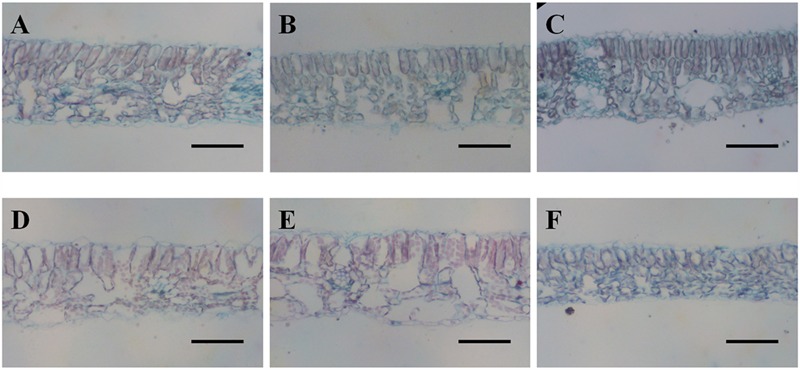
**Safranin-Fast Green staining of leaf blade sections from diploids (**A**, 80% FC; **B**, 50% FC; **C**, 20% FC) and hexaploids (**D**, 80% FC; **E**, 50% FC; **F**, 20% FC).** Scale bars are 50 μm.

**FIGURE 5:**
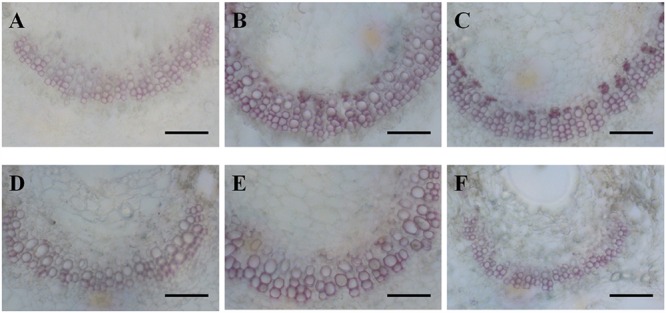
**Phloroglucinol-HCl staining of leaf midvein sections from diploids (**A**, 80% FC; **B**, 50% FC; **C**, 20% FC) and hexaploids (**D**, 80% FC; **E**, 50% FC; **F**, 20% FC).** Scale bars are 50 μm.

Cross sections of leaves indicated that the leaf blade was significantly thicker in diploid than in hexaploid plants because of the thicker palisade parenchyma under extreme water stress (**Table 3**; **Figure 4**). It is also worth noting that diploids always had a higher percentage of palisade parenchyma than hexaploids (**Table 3**; **Figure 4**). Consistent with the higher LMA observed for diploids under all soil water conditions and the compact cell packaging expected in leaves with higher LMA values, the palisade parenchyma cells in diploids were closely arranged, but those in hexaploids were more sparse (**Figure 4**). Hexaploids had significantly wider xylem conduits for low and medium stress and the xylem cell area in the leaf central vein was larger for diploids than that for hexaploids under severe water stress (**Table 3**; **Figure 5**).

**Table 3:** Anatomical characteristics of leaf blade and leaf central vein from diploid and hexaploid *C. angustifolium* under three different soil water treatments (80% of maximal FC, 50% FC, and 20% FC).

	Water treatment (% of maximum FC)		
	Low stress	Medium stress	High stress
	
Variable and cytotype	80% FC	50% FC	20% FC
**Leaf blade thickness (μm)**			
Diploid	78.972 ± 3.238 A,X	79.007 ± 4.074 A,X	83.120 ± 4.678 A,X
Hexaploid	78.688 ± 2.155 A,B,X	85.502 ± 4.325 A,X	70.637 ± 2.850 B,Y
**Palisade parenchyma thickness (μm)**			
Diploid	36.430 ± 1.822 A,X	35.942 ± 2.323 A,X	41.073 ± 2.710 A,X
Hexaploid	32.051 ± 0.619 A,Y	33.833 ± 1.737 A,X	30.591 ± 1.659 A,Y
**Lacunar parenchyma thickness (μm)**			
Diploid	36.218 ± 1.522 A,X	35.133 ± 1.804 A,X	33.131 ± 1.569 A,X
Hexaploid	39.511 ± 1.448 A,X	43.345 ± 2.550 A,Y	30.908 ± 1.738 B,X
**% of palisade thickness**			
Diploid	46.036 ± 0.914 A,X	45.218 ± 0.891 A,X	49.239 ± 1.085 B,X
Hexaploid	40.899 ± 0.967 A,B,Y	39.630 ± 0.803 A,Y	43.109 ± 0.842 B,Y
**Leaf central vein diameter (μm)**			
Diploid	387.829 ± 26.280 A,X	516.094 ± 20.449 B,X	625.681 ± 22.113 C,X
Hexaploid	533.085 ± 19.863 A,Y	684.340 ± 5.579 B,Y	385.444 ± 8.179 C,Y
**Xylem conduit diameter (μm)**			
Diploid	6.317 ± 0.176 A,X	8.070 ± 0.141 B,X	7.150 ± 0.201 C,X
Hexaploid	9.747 ± 0.327 A,Y	11.739 ± 0.306 B,Y	6.721 ± 0.229 C,X
**Leaf central vein total area (mm^2^)**			
Diploid	0.090 ± 0.012 A,X	0.208 ± 0.012 B,X	0.288 ± 0.028 C,X
Hexaploid	0.169 ± 0.016 A,Y	0.288 ± 0.016 B,Y	0.101 ± 0.009 C,Y
**Xylem cell area (mm^2^)**			
Diploid	0.011 ± 0.001 A,X	0.023 ± 0.002 B,X	0.025 ± 0.002 B,X
Hexaploid	0.012 ± 0.001 A,X	0.025 ± 0.001 B,X	0.008 ± 0.001 C,Y

*Each value represents mean ±*SE*. Letters after *SE* values distinguish between statistically separable (*P* < 0.05) values for different water treatments (A, B, C) and for different cytotypes (X, Y).*

## Discussion

In the Himalaya–Hengduan Mountains region, limited biogeographic studies manifested that diploid and derivative polyploids colonizing different distribution areas (Liang et al., 2015; Wu et al., 2016), with some polyploids tending to occur at high altitudes, such as *Aconitum* (Yuan and Yang, 2006), *Allium przewalskianum* (Cui et al., 2008), and *Anaphalis nepalensis* (Meng et al., 2014). Although geographical segregation of cytotypes has long been recognized, the underlying mechanisms creating these patterns remain poorly understood (McIntyre, 2012). Ecological sorting along environmental gradients and adaptive differences among ploidy levels may trigger habitat divergence (Duchoslav et al., 2010), a possibility which can be examined quantitatively by means of common garden and/or transplant experiments (McIntyre, 2012; Godsoe et al., 2013; Theodoridis et al., 2013; Glennon et al., 2014; McAllister et al., 2015). In our field investigations, we found that diploids occupied habitats at higher altitudes than hexaploids, supporting the previously reported pattern of distribution of different *C. angustifolium* cytotypes (Husband and Schemske, 1998). By performing a common garden experiment with different water stresses, we evaluated variations in drought tolerance traits in diploid and polyploid plants, and our results suggested that divergence in drought tolerance between polyploids and their diploid ancestors may have promoted habitat differentiation and the spatial separation of cytotypes.

### EcoPhysiological Differentiation between Ploidy Levels

Water availability as a growth limiting factor was demonstrated in the present study, since it caused significant reductions in TDM, RDM, and ADM, but the decreases in TDM and ADM in hexaploids were larger than those in diploids (**Table 1**). In addition, drought effects were more pronounced for above- than below-ground biomass in hexaploids, leading to an increase in R:S ratio, as would be generally expected (Fernandez and Reynolds, 2000; Otieno et al., 2005). Curiously, there was no detectable change in R:S ratio for diploid cytotype (**Figure 1**), indicating the existence of an intrinsic trait for coping with drought stress in diploid *C. angustifolium* (Ma et al., 2010; Ma et al., 2014). Plant growth is generally inhibited by water deficit before photosynthesis and maintenance of respiration, leading to the increase of non-structural carbon concentrations in tissues (Muller et al., 2011), which is commonly interpreted as osmotic adjustment (Hasibeder et al., 2015). During desiccation, parallel degree of osmotic adjustments-related assimilates allocation in roots and leaves may lead to the stable R:S ratio for perennial grasses to maintain water balance between organs, such as *Helianthus annuus* (Sobrado and Turner, 1986). This could explain the constant R:S ratio of diploids *C. angustifolium* across different gradients of water stress.

Drought can affect plant growth by influencing leaf gas exchange rates (Sapeta et al., 2013). Early responses to water deficit involve stomatal closure and a subsequent reduction in stomatal conductance (Chaves et al., 2002). The resulting reduction in leaf diffusive capacity then causes a simultaneous decline in CO_2_ uptake and transpiration during desiccation (Cornic and Massacci, 1996; Tezara et al., 1999). The magnitudes by which *g*_s_ was reduced (85.20% vs. 66.23%), *C*_i_ (25.24% vs. 15.88%), and *E* (74.86% vs. 56.06%) were greater in the hexaploids than in the diploids (**Table 1**). Lower *C*_i_ mediated by a reduction in leaf conductance may thus be inhibiting carbon metabolism in the face of drought stress (Downton et al., 1988; Maroco et al., 2002; Flexas et al., 2004) in both cytotypes (**Table 1**). Comparatively, diploids had a significantly higher *A*_max_ than hexaploids under severe water stress, and the reduction in *A*_max_ caused by drought in diploids (39.30%) was lower than that in hexaploids (53.94%; **Table 1**). Similarly, a higher photosynthetic rate during desiccation was found in diploids of *Mercurialis annua* (Buggs and Pannell, 2007) due to the case that polyploidy cytotype had lower transpiration rates and CO_2_ exchange rates under drought stress. Taking these results together, the fact that gas exchange was less depressed in diploids than in hexaploids would suggest that the former is better able to resist drought. Another reliable diagnostic indicator of plant photosynthetic activity is *F*_v_*/F*_m_ and its value often experiences a reduction under environmental stress (Maxwell and Johnson, 2000). Our results revealed that *F*_v_*/F*_m_ was significantly decreased by drought stress in hexaploids, to below the optimal value of 0.8 (**Table 1**) (Mena-Petite et al., 2000), indicating the sensitivity of hexaploids to drought stress.

WUE_i_ and WUE_L_ were enhanced from 50% FC to 80% FC by increasing drought stress for both cytotypes (**Table 1**; **Figure 2**); similar results have been reported in other species (Zhang and Marshall, 1994). Plants with high water use efficiency should have greater abilities to survive drought stress than those with lower water use efficiency (Lauteri et al., 1997). Our results indicated that WUE in diploids increased relative to that in hexaploids under severe water stress, reflecting a water use strategy adapted to growing in environments where drought stress was frequent.

The leaf is a major bottle-neck in the whole plant hydraulic conductivity system (Sack et al., 2003). Anatomical analysis revealed that diploid *C. angustifolium* possessed thicker leaves and palisade tissue (**Table 3**; **Figure 4**), and such morphological adjustments may enable the diploid to be more drought resistant than hexaploid (Li et al., 2009). Species that occur in dry areas are able to maintain living tissue at low RWC (Baltzer et al., 2008) and usually have thicker leaves (Rhizopoulou and Psaras, 2003). Accordingly, LMA tends to increase with leaf density and a high LMA is generally being considered as an adaptation to drought (Niinemets, 2001). Comparisons of LMA and RWC between ploidy levels showed that diploids had significantly higher LMA and lower RWC than hexaploids under extreme drought stress (**Table 1**; **Figure 3**), implying that the dense leaf tissues of diploids can withstand a low water content and thus slow water loss from the whole plant (Cunningham et al., 1999; Bucci et al., 2004).

### Tradeoff between Hydraulic Efficiency and Safety Associated with Genome Duplication

For hydraulic transport to be efficient, there should be a compromise between the ability to cope with water stress and the ability to grow at high rates under more favorable water conditions (Piñol and Sala, 2000; Martínez et al., 2002). As an instance, the lower xylem hydraulic conductivity found in higher ploidy levels of *Atriplex canescens* may be a major constraint counteracting the beneficial effects of their better drought tolerance (Hao et al., 2013). In contrast, the higher hydraulic conductivity of drought-sensitive diploid *A. canescens* may endow it with a higher growth rate (Stutz and Sanderson, 1983), and thus make it more competitive in environments with relatively high water availability (Sperry and Hacke, 2002). In *C. angustifolium*, higher hydraulic conductivity was detected in tetraploids than in diploids (Maherali et al., 2009). According to the above mentioned compromise rule, tetraploids may be much more drought-susceptible but competitive compared with diploids. Surprisingly, vulnerability to water stress induced cavitation was not found to differ across cytotypes (Maherali et al., 2009). The biomass of tetraploids was more negatively impacted by the imposition of water limitation than that of diploids, and both cytotypes had equal competitive abilities when water was limited (Thompson et al., 2015). Our experiments showed that both diploids and hexaploids of *C. angustifolium* were apparently affected by water shortage (as indicated by reductions in biomass accumulation and photosynthetic rate). Nevertheless, we found partial support for the hypothesis that diploids might be better able to cope with drought conditions than the polyploid cytotypes, since they showed physiological (e.g., gas exchange rate) and morphological (e.g., leaf architecture) adjustments appropriate for enduring water loss.

Both water deficit and freeze–thaw cycles can lead to xylem cavitation (Pockman and Sperry, 1996), and thus cytotypes with higher ploidy levels and larger conduits may be more vulnerable to drought stress due to the positive relationship between xylem conduit size and the risk of cavitation (Sperry and Sullivan, 1992; Hacke and Sperry, 2001; Martínez and Pockman, 2002). In *C. angustifolium*, polyploids generally had wider hydraulic vessel diameters than diploids (**Table 3**; **Figure 5**). Consequently, diploids, with their smaller xylem conduits, should be better able to avoid hydraulic disruption formed upon embolism (Pockman and Sperry, 1996), and a greater susceptibility to freezing-induced cavitation may exclude polyploids from sites at high altitudes (Maherali et al., 2009). In addition to the smaller xylem conduits, the vessel area of the leaf central vein in diploids was larger than that in hexaploids (**Table 3**; **Figure 5**), and the dense vascular bundle found in diploids was consistent with those observed in a few other cases (Maherali et al., 2009; Allario et al., 2011). A high density of major veins such as that found in diploids can provide a large number of parallel xylem pathways for water transport per leaf area, contributing to drought tolerance by routing water around embolized conduits (Scoffoni et al., 2011; Sack et al., 2012).

### Adaptive Significance of Polyploidization in Shaping Geographic Distribution

Two scenarios have been proposed to explain differences in patterns of cytotype distribution (Manzaneda et al., 2012). The adaptive evolutionary scenario suggests that shifts in ploidy level could result in differential stress tolerances (Levin, 2002; Baack, 2004; Kubátová et al., 2008; Sonnleitner et al., 2010; McAllister et al., 2015), which may underlie the ecological divergence and adaptation of cytotypes to novel environments (Ramsey, 2011). In contrast, the environmentally independent explanations (‘non-adaptive scenarios’) posit that exclusion of minority cytotypes (Levin, 1975) and historical processes (Sonnleitner et al., 2010; McAllister et al., 2015) may be the driving force behind the observed distribution patterns. Diploid *C. angustifolium* inhabits higher altitudes than polyploids, and a similar pattern has also been observed in *Senecio carniolicus* (Sonnleitner et al., 2010; Huelber et al., 2015) and *Centaurea jacea* (Hardy et al., 2000). The results of our study illustrate the distinct natures of physiological tolerance in the different ploidy levels, with diploids being less sensitive to drought stress than hexaploids. Thus, the occurrence of diploids in more open habitats at higher altitudes may be the result of adaptation that provides greater resistance to abiotic stress (Körner, 2003; Sonnleitner et al., 2010), whereas the predominance of polyploids in dense and nutrient-rich vegetation at lower altitudes may be due to greater competitiveness compared with the surrounding vegetation (Schönswetter et al., 2007; Ståhlberg, 2009). An earlier study indicated that differences in physiological tolerances in *C. angustifolium* probably evolved through natural selection acting on plant water relations after polyploidization (Maherali et al., 2009) and thus induced the adaptation of cytotypes to their native habitats (Martin and Husband, 2013). We therefore consider eco-physiological differentiation to be an important adaptive factor underlying the origin of the geographical separation and divergence in climatic niche (Thompson et al., 2014), although other, environmentally independent, factors cannot be excluded.

## Conclusion

In conclusion, our results suggest that drought endurance may have an important role in the segregation of *C. angustifolium* cytotypes across altitudinal gradients. Furthermore, genome duplication was suggested to provide the species with an opportunity to adapt to novel environments and thus to colonize new habitats (te Beest et al., 2011). We therefore tentatively suggest that cytotypes inhabiting high altitudes may be more tolerant to drought than those at low altitudes, independent of ploidy levels.

## Author Contributions

YWD, YPY, and WG designed the research and wrote the manuscript. WG, JY, XDS, and GJC performed experiments and conducted fieldwork.

## Conflict of Interest Statement

The authors declare that the research was conducted in the absence of any commercial or financial relationships that could be construed as a potential conflict of interest.
